# Aligning Horizon Scanning Approaches with Differing Needs and Topics at the Science–Policy Interface on Chemicals, Waste, and Pollution

**DOI:** 10.1021/acs.est.6c06644

**Published:** 2026-07-13

**Authors:** Gabriel Sigmund, Ricardo O. Barra, Michael G. Bertram, Miriam L. Diamond, Alex T. Ford, İpek İmamoğlu, Jinhui Li, Rainer Lohmann, Andreas Schäffer, Martin Scheringer, Kateřina Šebková, Maria Clara V. M. Starling, Noriyuki Suzuki, Penny Vlahos, Roland Weber, Bryan W. Brooks

**Affiliations:** † Environmental Technology, 4508 Wageningen University and Research, Wageningen 6700 AA, The Netherlands; ‡ Faculty of Environmental Sciences and EULA Chile Centre, University of Concepción, Concepción 4070386, Chile; § Department of Wildlife, 8095 Fish, and Environmental Studies, Swedish University of Agricultural Sciences, Umeå 907 36, Sweden; ∥ Department of Zoology, Stockholm University, Stockholm 114 18, Sweden; ⊥ School of Biological Sciences, Monash University, Melbourne 3800, Australia; # Department of Earth Sciences, 7938 University of Toronto, Toronto M5S 3B1, Canada; ¶ School of the Environment, University of Toronto, Toronto M5S 1A1, Canada; ∇ Institute of Marine Sciences, 6697 University of Portsmouth, Portsmouth PO4 9LY, U.K.; ○ Department of Environmental Engineering, 14643 Middle East Technical University, Ankara 06800, Turkey; ⧫ School of Environment, 12442 Tsinghua University, Haidian District, Beijing 100084, China; †† Graduate School of Oceanography, 54083 University of Rhode Island, Narragansett, Rhode Island 02882, United States; ‡‡ Institute for Environmental Research, RWTH Aachen University, Aachen 52074, Germany; §§ Department of Environmental Systems Science, ETH Zürich, Zürich 8092, Switzerland; ∥∥ RECETOX (Stockholm Convention Regional Centre), Faculty of Science, 602706 Masaryk University, Brno 62500, Czechia; ⊥⊥ Department of Sanitary and Environmental Engineering, 28114 Federal University of Minas Gerais, Belo Horizonte Minas Gerais 31270-010, Brazil; ## National Institute for Environmental Studies, Tsukuba 305-8506 Ibaraki, Japan; ¶¶ Department of Marine Sciences, University of Connecticut, Groton, Connecticut 06340, United States; ∇∇ POPs Environmental Consulting, Schwäbisch Gmünd 73527, Germany; ○○ Department of Environmental Science, Center for Reservoir and Aquatic Systems Research, Baylor University, Waco, Texas 76798, United States

**Keywords:** horizon scan, assessment, foresight, forecast, scenario, trends, science-policy

## Abstract

Knowledge on chemicals, waste, and pollution is shaped by geographical, financial, and disciplinary biases, which can cause blind spots for key emerging issues, including those relevant to low income countries and vulnerable communities. Scientists, practitioners, affected communities, and policy makers working in the areas of the newly established Intergovernmental Science-Policy Panel on Chemicals, Waste, and Pollution (ISP-CWP) have interest in identifying issues of potential and emerging relevance that currently escape their attention. Horizon scanning offers a critical tool to identify such issues. Here, we provide guidance on aligning horizon scanning approaches with differing objectives, audiences, and thematic scopes. We structure this guidance around three core dimensions: the topics addressed (“what”), the actors involved (“who”), and the methods applied (“how”). Drawing on existing horizon scanning efforts and foresight practices, we outline inclusive and transparent approaches suitable for prospective assessments across diverse contexts. Emphasis is placed on correcting epistemic asymmetries, integrating local and indigenous knowledge, and ensuring legitimacy for global governance processes. Strategically designed horizon scanning can support anticipatory policy, promote equity, and help steer collective action toward a livable planet for all.

## Graphical abstract

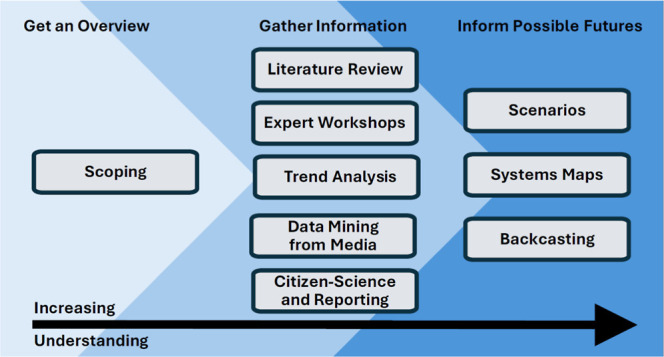

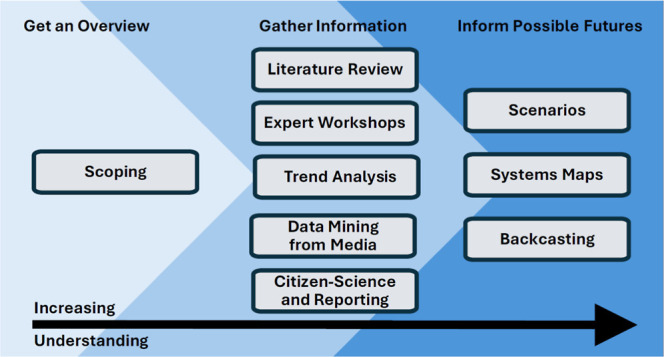

## Introduction

Scientific discourse is often biased toward subjects that are the most readily funded and most easily published.^1,2^ This bias prioritizes subjects and researchers in countries with adequate resources for research^3–5^ and favors investigating issues of concern to countries with adequate funding and research capacity.^4^ Scientists, practitioners, affected communities, and policy makers working in the areas of the newly established Intergovernmental Science-Policy Panel on Chemicals, Waste, and Pollution (ISP-CWP) have interest in identifying issues of potential and emerging relevance that may currently escape their attention. Horizon scanning can fill this gap by identifying potential issues based on current and emerging trends, serving to inform, caution against, suggesting future pathways, and countering “filters” that arise from these biases.

“Horizon scanning” is an umbrella term that covers a wide variety of approaches with different strengths and applicability domains that are intended to anticipate and explore future trajectories. A common objective of horizon scanning is not necessarily to predict the future, but to help decision makers identify and consider issues of concern and related possible actions with a certain degree of adaptability and flexibility. Currently, environmental governance in low- and middle-income countries (LMICs) tends to be reactive, focusing mainly on what is the most pressing problem at a given time, toward topics that are brought by multilateral environmental agreements and conventions ratified and/or following the lead of high income countries (HICs). The existing geographical and financial biases that prioritize the “hot topics” of HICs leave critical problems for LMICs on the periphery. Thus, to accurately reflect and address issues related to the global scope of chemicals, waste, and pollution, horizon scanning needs to inclusively also address issues that particularly affect underrepresented groups and regions, which are commonly overlooked by HICs and scientists therein. Integrating a situated perspective from LMICs is not only an act of inclusion but also a strategy to correct this epistemic asymmetry, ensuring that horizon scanning identifies risks that, although poorly illuminated by a lack of funding, may represent disproportionate global environmental burdens, especially for LMICs and vulnerable communities. Future priorities, for example within the ISP-CWP, may include themes that are prevalent in LMICs lacking ongoing monitoring and with limited research capacity; this could identify priority trends within and among locations and strategically shift resources to LMICs. In this respect, horizon scanning activities could catalyze action and awareness around poorly recognized issues for prospective assessments. Such efforts could help shed light on the unjust burden of global chemical and waste pollution, promoting living within safe and just earth system boundaries.^6,7^

Horizon scanning activities already have a track record of benefiting the science and practice on chemicals, waste, and pollution at global and regional levels. This is illustrated by HIC-driven future-focused activities, such as the World Economic Forum’s annual Global Risks Report,^8^ identifying Grand Challenges,^9^ and diverse contributions from the World Futures Studies Federation.^10^ Additional examples include routine efforts in conservation biology,^11^ initiatives by the Centers for Disease Control and Prevention,^12–14^ the Global Horizon Scanning project,^15–20^ the Needs for Onsite Wastewater Recycling Research Initiative,^21^ the planetary health check,^22^ and the recent Global Foresight Report on Planetary Health and Human Wellbeing by United Nations Environment Programme (UNEP).^23^ Other notable horizon scanning exercises focused on individual pollutants such as bisphenol A^24^ or diverse groups of chemicals such as pharmaceuticals.^25–28^ These examples illustrate that horizon scanning can help identify chemical-, waste-, and pollution-related threats, reveal management options and potential opportunities, and thus provide strategies and policy-relevant insights in HICs as well as LMICs. Therein, each horizon scanning effort targets different topics (the “what”) and different intended contributors and audiences (the “who”), which determine the approaches that are best suited to deliver a desired outcome (the “how”). Here, we provide an overview and guidance on these aspects with the intention to support the design of future horizon scanning activities on chemicals, waste, and pollution.

### The “What”

Conducting horizon scanning entails structured data generation and/or collection, analysis, and interpretation to forecast challenges and opportunities. Ideally, horizon scanning activities are transparent and inclusive. As efforts build up in response to societal awareness and the mandate of ISP-CWP, a culture of anticipation and preparedness is expected to develop.^29^ Here, horizon scanning would allow a systemic view of early warnings that could be picked up by a diverse group of constituencies.

For horizon scanning exercises, a clear and strategic problem statement is needed to direct the process. Such a statement should be tailored toward the desired outcome to guide the horizon scanning design and implementation based on specific interests and needs. For example, “How will sea level rise affect chemical emissions from landfill sites in coastal zones,” as sketched out in Diamond et al. (2024),^30^ is a specific problem statement with clear system boundaries that could guide the design of a related horizon scan. Thereby, different timelines and scopes need to be considered, in terms of impact and relevance on the immediate short-term as well as over longer time scales.^29^ Designing follow-up activities through time to identify how current topics are changing and new issues are emerging can be particularly useful, as evidenced in biological conservation, which recently reported findings from a 17th annual horizon scan.^31^

Sutherland and Woodroof (2009)^32^ caution against taking a “narrow forward look within a single domain;” this sage perspective is especially pertinent for the horizon scanning activities on chemicals, waste, and pollution, which are interdisciplinary issues in nature and affect and cross into other “domains” such as climate change and biodiversity loss.^11,33–35^ Thus, broad-scope horizon scanning activities could support integration across the three UN panels on climate change (IPCC), biodiversity loss (IPBES), and the ISP-CWP and reveal previously unrecognized interrelationships among the triple planetary crises.

### The “Who”

It is important to give agency to all interested parties, including often underrepresented groups such as LIC representatives, including scientists and civil society, to ensure that horizon scanning meets the goal of identifying early warning signals. Horizon scanning should be broadly inclusive of disciplines, each of which analyzes issues and solutions using a different and complementary lens.

In terms of “experts” to participate, corresponding and/or last authors from relevant scientific literature can be identified and engaged. However, this strategy can miss coauthors from LMICs who participate in HIC-driven studies. Moreover, particularly in LMICs, where peer-reviewed output and data coverage may be limited due to infrastructural and resource constraints, it is especially important to ensure an inclusive information gathering process. For example, a South African horizon scanning study to identify priority water research questions included more than two thousand participants from governmental institutions, private sector representatives, networks, and research community covering over two hundred organizations constituting an integrated, multidisciplinary, and inclusive effort.^36^ A collaborative network was formed by the use of a dedicated online platform from which participants contributed to surveys; later, selected specialists were invited to a workshop for prioritization of questions.^36,37^ Another recent large scale effort was conducted by the UNEP using an expert consultation and regional workshop to examine planetary health and well-being.^23,38^ Though a process overview was provided within this report, details related to how specific individuals were identified and how their inputs were solicited were not disclosed.

Recruitment of relevant participants for horizon scans may be achieved by, for instance, including civil servants and technical appointees, as well as country delegations, individuals, and groups involved in multilateral environmental agreements, such as the Basel, Rotterdam, Stockholm, and Minamata Conventions and the Montreal Protocol. Thereby, outputs of horizon scanning have improved chances of adoption by decision- and policy-makers if they are involved in its design and implementation, which is an aspect that should also be considered in the planning phase of any horizon scanning activity.^39^

Ensuring that horizon scanning is not an extractive data exercise but rather a process of participatory and equitable governance is necessary.^40^ Such activities could include regionally specific scoping based on local needs and priorities of affected communities, including Indigenous Peoples.^41^ For a horizon scan to have global legitimacy, it must grant real agency to all relevant actors, going beyond technical consultation with academic experts. Where scientific production may be limited by infrastructural constraints, local knowledge and the lived experiences of affected communities and Indigenous Peoples can be important sources of data. The inclusion of these voices makes it possible to detect “weak signals” of pollution such as transboundary movement of trade- and waste-related chemical pollution^42^ or the local impact and accumulation of pollution, which standardized models from HICs may miss.

Horizon scanning that includes regional workshops that periodically engage participants in a transparent and equitable manner could provide timely information such as science–policy implementation challenges and successes, technical and practice-focused education, training and research needs, and an opportunity for adaptive management which would be of particular value to the ISP-CWP. This approach should employ comprehensive and reliable horizon scanning methods for data collection, analysis, synthesis, prioritization, and/or reporting based on specific capacities and needs, as described below.

### The “How”

As described above, the selection and prioritization of themes and specific topics for Horizon Scanning activities on chemicals, waste, and pollution and as part of the ISP-CWP’s outputs will need to be operationalized, taking into account the wide subject scope.^30^ Performing these analyses will require a systematic approach and reliable data. However, even when available, often data are not collected, maintained, and shared consistently across United Nations member states. In this regard, “best practice” guidelines should be developed for ISP-CWP members and the broader community to generate FAIR (findable, accessible, interoperable, and reusable) records to make future assessments and reporting more efficient and abide by CARE principles (Collective Benefit, Authority to control, Responsibility, Ethics) for indigenous data. This is critical to setting a foundational base and establishing effective partnerships for any assessment, including horizon scans. Approaches to validate and verify evidence deriving from diverse knowledge streams, such as “gray literature,” local knowledge, lived experiences, and media-based signals, will need to be developed to ensure credibility and trust in the legitimacy of horizon scan findings. Similarly, a transparent and harmonized approach to convey varying degrees of (un)­certainty in prospective assessments must also be developed. The resulting compilation of FAIR information and data will require substantial infrastructure and support for those contributing to this effort.

Sutherland and Woodroof (2009)^32^ and Sutherland et al. (2011)^43^ summarized common horizon scanning methods and approaches, which range from critical reviews and trend analyses to expert workshops and open source data collection. Horizon scanning approaches were categorized based on their “scanning stage,” which relates to the maturity of the understanding for a given issue. Approaches related to this sequence of increasing understanding are discussed below. Figure 1 shows how these approaches can complement and inform each other.

**Figure 1:**
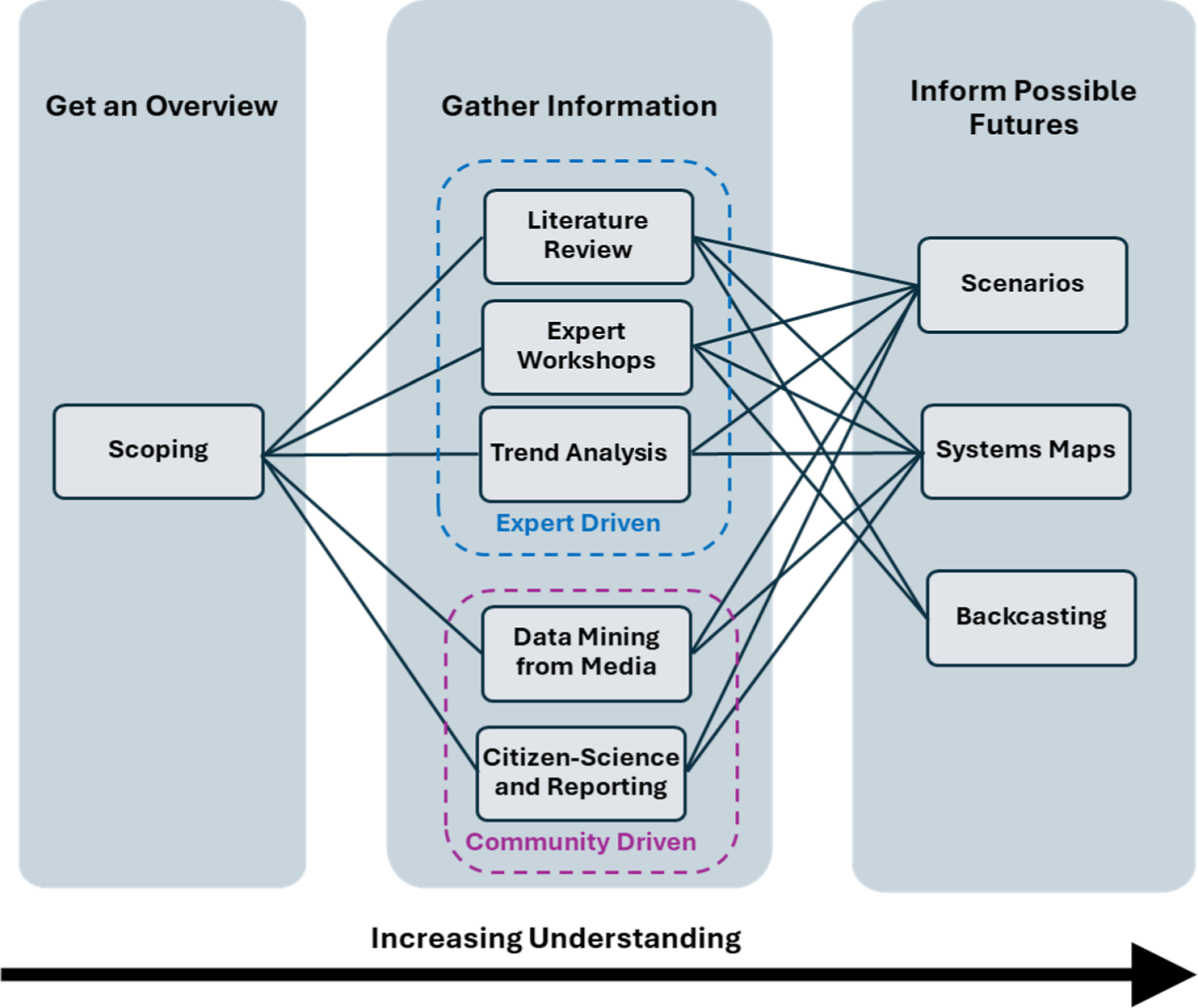
An example of horizon scanning approaches with increasing understanding.

### Scoping

To inform horizon scanning priorities and subsequent activities at the science–policy interface of chemicals, waste, and pollution, a transparent and open procedure needs to be developed to collect topics of interest, which are then prioritized based on set criteria. Specific and prioritized topics could then be scoped using an “issue tree.” This approach breaks down key questions into a mutually exclusive and completely exhaustive set of sub-questions. Thereafter, it identifies the information needed to provide a complete answer to key questions.^32^ This approach is suitable for specific and well-defined issues and was used, for example, in the Foresight project on Brain Science, Addiction, and Drugs.^44^ More generic and less precisely defined topics are not well-suited for this approach and may be scoped using interviews with key experts and diverse knowledge holders, taking into account representative backgrounds and lived experiences, to then potentially define a more specific sub-question to explore with an issue tree.

### Gathering Information

One of the most common approaches to gathering information relating to specific questions is to conduct systematic (peer-reviewed) literature reviews that are suitable for the retrospective assessment of topics that are well-researched and documented. Therein, adequate quality control should be established by subject experts, e.g., for experimental work including considerations on controls, replicates, and the relevance of experimental conditions should be considered for ranking studies that are considered. An important aspect to consider when applying such approaches is the implicit bias toward topics and institutions who have the capacity to publish in peer-reviewed journals, especially when considering high-impact journals which are typically weighted more strongly than other publications. Thus, key issues and insights from LMICs, non-academic knowledge holders, and Indigenous Peoples are inherently underrepresented. In contrast, expert workshops can be designed to be more inclusive to a diversity of experts and underrepresented groups, regional backgrounds, and lived experiences while maintaining data quality standards and credibility. Additionally, expert workshops can provide space for novel and creative ideas to reframe topics collectively. This approach also allows for the question-dependent tailoring of the “who” discussed above. Hybrid in-person and online formats allow for diverse participation less apt to restriction according to financial resources.

In addition to these expert-centric approaches, more decentralized bottom-up approaches may also be suitable for the gathering of information pertaining to specific topics. Such approaches include the following:

(i) The collection and mining of data from traditional and new media, including crowd-sourced information, e.g., for environmental pollution events, observed health effects, and public attention on specific issues, could be performed in a systematic and harmonized manner. The specific type of desired information, the question, or the primary purpose will dictate the process, method, and practice. Scanning local news in all languages, potentially using AI-based language models for harmonized translation, could help to avoid biases against key issues for HICs, which is common with peer-reviewed papers.^45^ Therein, a reliable and well-documented methodology to filter such media will need to be developed and agreed upon to avoid fake news and misinformation. Similarly, standards need to be established to ensure that AI-based models are trained on appropriate and unbiased data.
(ii) Disaster reporting and communication mechanisms are also useful model templates to consider. For example, app technologies such as the Disaster Alert app (https://disasteralert.pdc.org/disasteralert/) that provides near real-time alerts and also incorporates user-reported data on volcanoes, wildfires, floods, etc., which could form the basis of, e.g., trend analysis (see later). Similar apps that enable citizen science and involvement for reporting topics of interest to the ISP-CWP could be considered on a need basis. In addition, individual-reported data through social media platforms (e.g., “Marked Safe” in Facebook) and roadway hazard reporting by drivers (e.g., Apple and Google Maps) are commonly used by the public. They could provide useful citizen-science-based and whistle blower approaches for the future development of information crowdsourcing in an inclusive and decentralized manner. Such approaches, which should include a secure and independent process to vet such reports and protect sources, could provide spatiotemporally resolved data that can inform horizon scanning in support of the ISP-CWP. For example, local fishermen initially identified fish kills, which informed government intervention of cyanide discharges from a large steel plant.^46,47^ Similar approaches are deployed by citizen-science platforms across a range of end points, including air pollution.^48^ We recognize that such apps and platforms may not be available or safe in certain countries.
(iii) Consultation of experts through questionnaires, including follow-up questions, is a common approach for providing an overview of specific areas of science, as illustrated by a recent Delphi study on the key concerns surrounding persistent chemicals,^49^ as well as another study on “Chemical Pollution–Related Policymaking for Sustainable and Climate-Resilient Economies.”^50^

### Spotting Signals and Watching Trends

Trend analysis is used to forecast future trends based on past performance. This approach is useful to identify and understand past drivers. However, past trends and drivers may not necessarily provide insight in an ever changing future, and related outcomes are inherently uncertain. A prominent example for such an approach is the State of the Nation’s Ecosystems 2008.^51^ Importantly, impact- and risk-based approaches are mostly inadequate for prospective assessments and forecasting exercises, as the related knowledge base is typically insufficient for emerging issues and topics in the ISP-CWP sphere. Therefore, in most cases, it is advisable to base forecasting on hazards, for which knowledge is more readily available.^52^ Hazard-based approaches also tend to be more generalizable than impact- or risk-based approaches, which are geospatially situational by design.

### Making Sense of and Agreeing with the Response

Considering possible future states to explore potential consequences of defined scenarios can be a powerful tool for forecasting the effect of planned measures. Such activities are well-documented and known from the scenario-based reports of the IPCC.^53^ However, these types of assessments require a substantial amount of resources to be developed, in terms of both expertise and time. Thus, it is sensible to only conduct scenario-based analysis for a select number of topics based on priority and assessment capacity.

Systems maps can illustrate relationships among factors influencing a defined central issue. These exercises can provide an understanding of a range of issues influencing the central issue and inform prioritization of, e.g., mitigation measures to tackle the central issue at hand. System mapping is needed to understand interlocking systems and to avoid a reductionist analysis that could lead to unintended consequences, including undesirable risk tradeoffs, particularly in LMICs and underserved and disproportionately affected communities of HICs. Similarly to scenario-based assessments, system maps require in-depth subject knowledge and sufficient time for development. For example, such an approach was used to assess the perceived relevance and organizational support for “Agents of Change” for sustainable land management in agriculture in Aotearoa New Zealand.^54^ “Theory of Change” is another similar analytical tool that provides insights into a solution or intervention by explicitly considering connections between an intended intervention and intended outcomes.^55^ In addition to their use by “experts,” system maps and Theories of Change provide a focal point for stakeholder consultations.

Backcasting approaches are based on the description of a desired vision of the future and the subsequent identification of the key steps necessary to reach that vision. As such, they can be useful catalyzers to compare, prioritize, and motivate solution-oriented assessments. Recent examples for ISP-CWP-related backcasting activities include future visions for bioplastics substituting PET^56^ and the integration of the Polyurethane Foam Industry into a more circular economy.^57^

### A Horizon of Opportunities

In a time of multiple environmental crises, geopolitical uncertainty, and tension, prospective and inclusive assessments can inform feasible paths forward. Now, more than ever, multilateral efforts are needed to generate co-benefits for those most affected by these crises, including LMICs, vulnerable local communities, and Indigenous Peoples. While institutional progress may be slow and face a number of situational obstacles, horizon scanning in the spirit of and by the ISP-CWP offers the opportunity to synthesize knowledge and inform paths forward. Inclusive, transparent, and cross-sectoral ambitious prospective assessments are urgently needed to inform policy. Now is the time for the scientific community to step up to this challenge and deliver on the promise of collective progress and cooperation toward a livable planet for all. The urgency is on us, as the next decades will force human societies to adapt to a rapidly changing planet facing triple planetary crises that will challenge us all.^6,7,58^

## References

[1] Kristiansson E., Coria J., Gunnarsson L., Gustavsson M. (2021). Does the Scientific
Knowledge Reflect the Chemical Diversity of Environmental Pollution?
– A Twenty-Year Perspective. Environ.
Sci. Policy.

[2] Sobek A., Bejgarn S., Rudén C., Breitholtz M. (2016). The Dilemma
in Prioritizing Chemicals for Environmental Analysis: Known versus
Unknown Hazards. Environ. Sci. Process. Impacts.

[3] Chen C. Y., Kahanamoku S. S., Tripati A., Alegado R. A., Morris V. R., Andrade K., Hosbey J. (2022). Systemic Racial Disparities in Funding
Rates at the National Science Foundation. eLife.

[4] Diamond M. L., Rosario-Ortiz F., Field J., Leusch F., Lowry G., Mills M., Wang P., Westerhoff P., Zimmerman J. (2022). Hearing All Voices to Address Environmental Challenges
at a Global Scale. Environ. Sci. Technol..

[5] Heesen R., Bright L. K. (2025). Publication Bias
Is Bad for Science If Not Necessarily
Scientists. R. Soc. Open Sci..

[6] Rockström J., Gupta J., Qin D., Lade S. J., Abrams J. F., Andersen L. S., Armstrong McKay D. I., Bai X., Bala G., Bunn S. E., Ciobanu D., DeClerck F., Ebi K., Gifford L., Gordon C., Hasan S., Kanie N., Lenton T. M., Loriani S., Liverman D. M., Mohamed A., Nakicenovic N., Obura D., Ospina D., Prodani K., Rammelt C., Sakschewski B., Scholtens J., Stewart-Koster B., Tharammal T., van Vuuren D., Verburg P. H., Winkelmann R., Zimm C., Bennett E. M., Bringezu S., Broadgate W., Green P. A., Huang L., Jacobson L., Ndehedehe C., Pedde S., Rocha J., Scheffer M., Schulte-Uebbing L., de Vries W., Xiao C., Xu C., Xu X., Zafra-Calvo N., Zhang X. (2023). Safe and Just Earth
System Boundaries. Nature.

[7] Diamond M. L., Wang Z. (2024). Safe and Just Earth
System Boundaries for Novel Entities. Environ.
Sci. Technol..

[8] Elsner, M. ; Atkinson, G. ; Zahidi, S. Global Risks Report 2025; World Economic Forum, 2025. https://www.weforum.org/publications/global-risks-report-2025/ (accessed July 7, 2026).

[9] National Academy of Engeneering . NAE Grand Challenges for Engineering; National Academy of Engeneering, 2017. https://www.nae.edu/187212/NAE-Grand-Challenges-for-Engineering (accessed July 7, 2026).

[10] Futures Publications – Journals – WORLD FUTURES STUDIES FEDERATION. https://wfsf.org/futures-publications-journals/ (accessed July 7, 2026).

[11] Sutherland W. J., Adams W. M., Aronson R. B., Aveling R., Blackburn T. M., Broad S., Ceballos G., Côté I. M., Cowling R. M., Da Fonseca G. a. B., Dinerstein E., Ferraro P. J., Fleishman E., Gascon C., Hunter M., Hutton J., Kareiva P., Kuria A., Macdonald D. W., Mackinnon K., Madgwick F. J., Mascia M. B., Mcneely J., Milner-Gulland E. J., Moon S., Morley C. G., Nelson S., Osborn D., Pai M., Parsons E. C. M., Peck L. S., Possingham H., Prior S. V., Pullin A. S., Rands M. R. W., Ranganathan J., Redford K. H., Rodriguez J. P., Seymour F., Sobel J., Sodhi N. S., Stott A., Vance-Borland K., Watkinson A. R. (2009). One Hundred Questions of Importance
to the Conservation of Global Biological Diversity. Conserv. Biol..

[12] Gerding J. A., Landeen E., Kelly K. R., Whitehead S., Dyjack D. T., Sarisky J., Brooks B. W. (2019). Uncovering
Environmental
Health: An Initial Assessment of the Profession’s Health Department
Workforce and Practice. J. Environ. Health.

[13] Brooks B.
W., Gerding J. A., Landeen E., Bradley E., Callahan T., Cushing S., Hailu F., Hall N., Hatch T., Jurries S., Kalis M. A., Kelly K. R., Laco J. P., Lemin N., McInnes C., Olsen G., Stratman R., White C., Wille S., Sarisky J. (2019). Environmental Health
Practice Challenges and Research Needs for U.S. Health Departments. Environ. Health Perspect..

[14] Gerding J. A., Brooks B. W., Landeen E., Whitehead S., Kelly K. R., Allen A., Banaszynski D., Dorshorst M., Drager L., Eshenaur T., Freund J., Inman A., Long S., Maloney J., McKeever T., Pigman T., Rising N., Scanlan S., Scott J., Shukie C., Stewart G., Tamekazu D., Wade V., White C., Sarisky J. (2020). Identifying Needs for Advancing the
Profession and Workforce in Environmental Health. Am. J. Public Health.

[15] Brooks B. W., Ankley G. T., Boxall A. B., Rudd M. A. (2013). Toward
Sustainable
Environmental Quality: A Call to Prioritize Global Research Needs. Integr. Environ. Assess. Manage..

[16] Furley T. H., Brodeur J., Silva de Assis H. C., Carriquiriborde P., Chagas K. R., Corrales J., Denadai M., Fuchs J., Mascarenhas R., Miglioranza K. S., Miguez Caramés D. M., Navas J. M., Nugegoda D., Planes E., Rodriguez-Jorquera I. A., Orozco-Medina M., Boxall A. B., Rudd M. A., Brooks B. W. (2018). Toward
Sustainable Environmental Quality: Identifying Priority Research Questions
for Latin America. Integr. Environ. Assess.
Manage..

[17] Van
den Brink P. J., Boxall A. B. A., Maltby L., Brooks B. W., Rudd M. A., Backhaus T., Spurgeon D., Verougstraete V., Ajao C., Ankley G. T., Apitz S. E., Arnold K., Brodin T., Cañedo-Argüelles M., Chapman J., Corrales J., Coutellec M., Fernandes T. F., Fick J., Ford A. T., Giménez
Papiol G., Groh K. J., Hutchinson T. H., Kruger H., Kukkonen J. V. K., Loutseti S., Marshall S., Muir D., Ortiz-Santaliestra M.
E., Paul K. B., Rico A., Rodea-Palomares I., Römbke J., Rydberg T., Segner H., Smit M., van Gestel C. A. M., Vighi M., Werner I., Zimmer E. I., van Wensem J. (2018). Toward Sustainable
Environmental Quality: Priority Research Questions for Europe. Environ. Toxicol. Chem..

[18] Fairbrother A., Muir D., Solomon K. R., Ankley G. T., Rudd M. A., Boxall A. B. A., Apell J. N., Armbrust K. L., Blalock B. J., Bowman S. R., Campbell L. M., Cobb G. P., Connors K. A., Dreier D. A., Evans M. S., Henry C. J., Hoke R. A., Houde M., Klaine S. J., Klaper R. D., Kullik S. A., Lanno R. P., Meyer C., Ottinger M. A., Oziolor E., Petersen E. J., Poynton H. C., Rice P. J., Rodriguez-Fuentes G., Samel A., Shaw J. R., Steevens J. A., Verslycke T. A., Vidal-Dorsch D. E., Weir S. M., Wilson P., Brooks B. W. (2019). Toward
Sustainable Environmental Quality: Priority Research Questions for
North America. Environ. Toxicol. Chem..

[19] Gaw S., Harford A., Pettigrove V., Sevicke-Jones G., Manning T., Ataria J., Cresswell T., Dafforn K. A., Leusch F. D., Moggridge B., Cameron M., Chapman J., Coates G., Colville A., Death C., Hageman K., Hassell K., Hoak M., Gadd J., Jolley D. F., Karami A., Kotzakoulakis K., Lim R., McRae N., Metzeling L., Mooney T., Myers J., Pearson A., Saaristo M., Sharley D., Stuthe J., Sutherland O., Thomas O., Tremblay L., Wood W., Boxall A. B., Rudd M. A., Brooks B. W. (2019). Towards Sustainable
Environmental Quality: Priority Research Questions for the Australasian
Region of Oceania. Integr. Environ. Assess.
Manage..

[20] Leung K. M. Y., Yeung K. W. Y., You J., Choi K., Zhang X., Smith R., Zhou G., Yung M. M. N., Arias-Barreiro C., An Y., Burket S. R., Dwyer R., Goodkin N., Hii Y. S., Hoang T., Humphrey C., Iwai C. B., Jeong S., Juhel G., Karami A., Kyriazi-Huber K., Lee K., Lin B., Lu B., Martin P., Nillos M. G., Oginawati K., Rathnayake I. V. N., Risjani Y., Shoeb M., Tan C. H., Tsuchiya M. C., Ankley G. T., Boxall A. B. A., Rudd M. A., Brooks B. W. (2020). Toward Sustainable Environmental
Quality: Priority Research Questions for Asia. Environ. Toxicol. Chem..

[21] Brooks B. W., Callahan T., Stanley J. K., Holodak J., Stroski K. M., Cox A. H., Groves T. W., Jantrania A., Moeller J. C., Neset K., Walker C., Zhang H., Bakchan A., Alley K. D., Bell J., Blodig A., Casey E., Cosper D., D’Amato V., Elliott M. A., Graves G., Groover R., Himschoot R., Lusk M. G., Maxcy-Brown J., Meints D., Mejia R., Pace M., Ryan B. J., Scheffe B., Schimelfenig T., Wolfe J. E., Heger S. F. (2026). Identifying Priority Research Questions
for Decentralized Wastewater. Environ. Sci.
Technol..

[22] Planetary Health Check . Planetary Health Check. https://www.planetaryhealthcheck.org/ (accessed July 7, 2026).

[23] UNEP . Navigating New Horizons: A Global Foresight Report on Planetary Health and Human Wellbeing; United Nations Environment Programme, 2024. https://wedocs.unep.org/items/6377804d-7311-45f0-8564-8965452e343a (accessed July 7, 2026).

[24] Corrales J., Kristofco L. A., Steele W. B., Yates B. S., Breed C. S., Williams E. S., Brooks B. W. (2015). Global Assessment of Bisphenol A
in the Environment: Review and Analysis of Its Occurrence and Bioaccumulation. Dose-Response.

[25] Boxall A. B. A., Rudd M. A., Brooks B. W., Caldwell D. J., Choi K., Hickmann S., Innes E., Ostapyk K., Staveley J. P., Verslycke T., Ankley G. T., Beazley K. F., Belanger S. E., Berninger J. P., Carriquiriborde P., Coors A., DeLeo P. C., Dyer S. D., Ericson J. F., Gagné F., Giesy J. P., Gouin T., Hallstrom L., Karlsson M. V., Larsson D. G. J., Lazorchak J. M., Mastrocco F., McLaughlin A., McMaster M. E., Meyerhoff R. D., Moore R., Parrott J. L., Snape J. R., Murray-Smith R., Servos M. R., Sibley P. K., Straub J. O., Szabo N. D., Topp E., Tetreault G. R., Trudeau V. L., Van Der
Kraak G. (2012). Pharmaceuticals and Personal Care Products in the Environment: What
Are the Big Questions?. Environ. Health Perspect..

[26] Rudd M. A., Ankley G. T., Boxall A. B., Brooks B. W. (2014). International Scientists’
Priorities for Research on Pharmaceutical and Personal Care Products
in the Environment. Integr. Environ. Assess.
Manage..

[27] Boxall A. B. A., Brooks B. W. (2024). Pharmaceuticals
and Personal Care Products in the Environment:
What Progress Has Been Made in Addressing the Big Research Questions?. Environ. Toxicol. Chem..

[28] Kristofco L. A., Brooks B. W. (2017). Global Scanning of Antihistamines in the Environment:
Analysis of Occurrence and Hazards in Aquatic Systems. Sci. Total Environ..

[29] European Environment Agency Horizon Scanning - Tips and Tricks A Practical Guide, 2023; . 10.2800/360744.

[30] Diamond M. L., Sigmund G., Bertram M. G., Ford A. T., Ågerstrand M., Carlini G., Lohmann R., Šebková K., Soehl A., Starling M. C. V. M., Suzuki N., Venier M., Vlahos P., Scheringer M. (2024). Exploring
Outputs of the Intergovernmental
Science-Policy Panel on Chemicals, Waste, and Pollution Prevention. Environ. Sci. Technol. Lett..

[31] Sutherland W. J., Butchart S. H. M., Clarke S. J., Doar N. R., Doran H., Douglas I. C., Field D. J., Fleishman E., Gaston K. J., Herbert-Read J. E., Hughes A. C., Kaartokallio H., Maggs L., Palardy J. E., Pearce-Higgins J. W., Peck L. S., Pettorelli N., Schloss I. R., Spalding M. D., Timoshyna A., Tubbs N., Uehara T., Watson J. E. M., Wentworth J., Wilson J. D., Thornton A. (2026). A Horizon Scan of Biological
Conservation Issues for 2026. Trends Ecol. Evol..

[32] Sutherland W. J., Woodroof H. J. (2009). The Need for Environmental
Horizon Scanning. Trends Ecol. Evol..

[33] Sigmund G., Ågerstrand M., Antonelli A., Backhaus T., Brodin T., Diamond M. L., Erdelen W. R., Evers D. C., Hofmann T., Hueffer T., Lai A., Torres J. P. M., Mueller L., Perrigo A. L., Rillig M. C., Schaeffer A., Scheringer M., Schirmer K., Tlili A., Soehl A., Triebskorn R., Vlahos P., vom Berg C., Wang Z., Groh K. J. (2023). Addressing Chemical Pollution in
Biodiversity Research. Glob. Change Biol..

[34] Baste I. A., Watson R. T. (2022). Tackling the Climate,
Biodiversity and Pollution Emergencies
by Making Peace with Nature 50 Years after the Stockholm Conference. Glob. Environ. Change.

[35] Bălan S. A., van Bergen S. K., Blake A., Buck T., Coffin S., DeWitt J. C., Goldenman G., von Hippel F. A., von Hippel S., Leonetti C. P., Rist D., Scheringer M., Trier X. (2025). Confronting the Interconnection of Chemical Pollution and Climate
Change. Environ. Innov. Soc. Transit..

[36] Siebrits R. M., Winter K., Barnes J., Dent M. C., Ginster M., Harrison J., Jackson B., Jacobs I., Jordaan A., Kasan H. C., Kloppers W., Le Roux R., Maree J., Momba M. N. B., Munnik A. V., O’Keeffe J., Schulze R., Silberbauer M., Still D., Van Zyl J. (2014). Priority Water
Research Questions for South Africa Developed through Participatory
Processes. Water SA.

[37] Siebrits R., Winter K., Jacobs I. (2014). Water Research Paradigm
Shifts in
South Africa. South Afr. J. Sci..

[38] Jabbour J., Davidson D. J., Carlsen H., King N., Lucatello S., Aricò S., Cooke F. L., Datta R., Gluckman P., Gutierrez-Espeleta E.
E., Hinwood A., Jia G., Komendantova N., Lunga W., Madise N., Mangalagiu D., Ali E. M., Moronta-Barrios F., Mycoo M., Piyawatanametha W., Stevance A.-S., Swaminathan S. M., Ürge-Vorsatz D., Bojic L. (2026). Navigating the Winds of Change: Strategic Foresight and the Power
of Weak Signals. Sustain. Sci..

[39] Bierbaum R., Leonard S. A., Rejeski D., Whaley C., Barra R. O., Libre C. (2020). Novel Entities and
Technologies: Environmental Benefits and Risks. Environ. Sci. Policy.

[40] Dorji T., Rinchen K., Morrison-Saunders A., Blake D., Banham V., Pelden S. (2024). Understanding How Indigenous
Knowledge Contributes
to Climate Change Adaptation and Resilience: A Systematic Literature
Review. Environ. Manage..

[41] Ataria J. M., Murphy M., McGregor D., Chiblow S., Moggridge B. J., Hikuroa D. C. H., Tremblay L. A., Öberg G., Baker V., Brooks B. W. (2023). Orienting the Sustainable
Management
of Chemicals and Waste toward Indigenous Knowledge. Environ. Sci. Technol..

[42] Wania F., Zhan F., Chen C., Solano Diaz K., Ai Z., Kasperkiewicz A., Glienke J. (2026). Toxic Organic Chemicals
in a Globally Connected World: Comparing Real Flows with Virtual Flows
Embodied in International Trade. Environ. Sci.
Technol..

[43] Sutherland W. J., Fleishman E., Mascia M. B., Pretty J., Rudd M. A. (2011). Methods
for Collaboratively Identifying Research Priorities and Emerging Issues
in Science and Policy. Methods Ecol. Evol..

[44] Nutt D. J. (2005). Foresight
Brain Science, Addiction and Drugs Project. J. Psychopharmacol. (Oxf.).

[45] National Data on Public Health Workforce | PH WINS. https://www.phwins.org/dashboard (accessed July 7, 2026).

[46] Press, A. Vietnam Blames Toxic Waste Water from Steel Plant for Mass Fish Deaths. The Guardian July 1, 2016. https://www.theguardian.com/environment/2016/jul/01/vietnam-blames-toxic-waste-water-fom-steel-plant-for-mass-fish-deaths (accessed July 7, 2026).

[47] France-Presse, A. Vietnam Investigates Mass Fish Deaths. The Guardian. April 21, 2016. https://www.theguardian.com/environment/2016/apr/21/vietnam-investigates-mass-fish-deaths-pollution (accessed July 7, 2026).

[48] Apps - SPOTTERON Citizen Science. https://www.spotteron.net/apps (accessed July 7, 2026).

[49] Thiele K., Tobi H., Gabbert S. (2025). What’s
the Concern about Persistent
Chemicals? Insights from a Delphi Study. Environ.
Sci. Policy.

[50] Green C., Bilyanska A., Bradley M., Dinsdale J., Hutt L., Backhaus T., Boons F., Bott D., Collins C., Cornell S. E., Craig M., Depledge M., Diderich B., Fuller R., Galloway T. S., Hutchison G. R., Ingrey N., Johnson A. C., Kupka R., Matthiessen P., Oliver R., Owen S., Owens S., Pickett J., Robinson S., Sims K., Smith P., Sumpter J. P., Tretsiakova-McNally S., Wang M., Welton T., Willis K. J., Lynch I. (2023). A Horizon
Scan to Support Chemical Pollution–Related Policymaking
for Sustainable and Climate-Resilient Economies. Environ. Toxicol. Chem..

[51] John, H. Heinz III Center for Science, Economics and the Environment. The State of the Nation’s Ecosystems 2008: Measuring the Lands, Waters, and Living Resources of the United States 2008. https://www.aei.org/research-products/report/the-state-of-the-nations-ecosystems/ (accessed July 7, 2026).

[52] European Environment Agency . Late Lessons from Early Warnings: Science, Precaution, Innovation; Publication; 2013. https://www.eea.europa.eu/publications/late-lessons-2 (accessed July 7, 2026).

[53] Special Report on Emissions Scenarios: A Special Report of Working Group III of the Intergovernmental Panel on Climate Change, 1. Publ.; NakićEnović, N., IPCC, Eds.; Cambridge University Press: Cambridge, 2000.

[54] Small B., Berg N., Connolly J. D., Cunningham M. F. W., Harcourt N., Kirk N., McFetridge A., McLachlan S., van Noppen F. D., Robson-Williams M. (2026). Leveraging
Change: Perceived Relevance and Organisational Support for Agents
of Change for Sustainable Land Management in Agriculture in Aotearoa
New Zealand. Ko̅tuitui N. Z. J. Soc. Sci.
Online.

[55] Unsdg | UNDAF. Guidance on Theory of Change; 2016. https://unsdg.un.org/resources/undaf-guidance-theory-change (accessed July 7, 2026).

[56] Haas V., Wenger J., Ranacher L., Guigo N., Sousa A. F., Stern T. (2022). Developing Future Visions
for Bio-Plastics Substituting PET –
A Backcasting Approach. Sustain. Prod. Consum..

[57] Abadian, M. ; Russell, J. D. Exploring Backcasting as a Tool to Co-Create a Vision for a Circular Economy: A Case Study of the Polyurethane Foam Industry. J. Circ. Econ. 2024, 2 2 10.55845/UZXQ5070.

[58] OECD . Roadmap for Policy Action: Environmental Outlook on the Triple Planetary Crisis; 2025. https://www.oecd.org/en/publications/environmental-outlook-on-the-triple-planetary-crisis_257ffbb6-en/full-report/roadmap-for-policy-action_b27692cb.html (accessed July 7, 2026).

